# Increased empathic distress in adults is associated with higher levels of childhood maltreatment

**DOI:** 10.1038/s41598-023-30891-7

**Published:** 2023-03-11

**Authors:** Annika B. E. Benz, Stephanie J. Dimitroff, Christin Jeggle, Raphaela J. Gaertner, Maria Meier, Eva Unternaehrer, Ulrike U. Bentele, Bernadette F. Denk, Elea S. C. Klink, Jens C. Pruessner

**Affiliations:** 1 Division of Neuropsychology, Department of Psychology, University of Konstanz, Fach 905, Universitaetsstrasse 10, 78464 Constance, Germany; 2 Present Address: Child- and Adolescent Research Department, Psychiatric University Hospitals Basel (UPK), University of Basel, Basel, Switzerland; 3 Centre for the Advanced Study of Collective Behaviour, University of Constance, Constance, Germany

**Keywords:** Psychology, Human behaviour

## Abstract

While many studies investigated basic facets of empathy, less is known about the association with early life adversity (ELA). To investigate a possible association of empathy with ELA, we assessed self-reported ELA, using the Childhood Trauma Questionnaire (CTQ), the Parental Bonding Instrument (PBI) for mother and father, and empathy, using the Interpersonal Reactivity Index (IRI), in a sample of *N* = 228 (83% female, age_mean_ = 30.51 ± 9.88 years, age_range_ = 18–60). Further, we measured willingness to donate a certain percentage of study compensation to a charity as an index of prosocial behavior. In line with our hypotheses that stated a positive association of empathy with ELA, increased levels of emotional, physical, and sexual abuse, and emotional and physical neglect were positively correlated with personal distress in response to others’ suffering. Likewise, higher parental overprotection and lower parental care were related to higher personal distress. Furthermore, while participants with higher levels of ELA tended to donate more money on a merely descriptive level, only higher levels of sexual abuse were significantly related to larger donations after correction for multiple statistical tests. Other facets of the IRI (empathic concern, perspective taking and fantasy) were not related to any other ELA measure. This suggests ELA only affects levels of personal distress.

## Introduction

Empathy is a complex, and malleable social phenomenon that allows us to gain insights into the thoughts and feelings of others^1–3^. However, feelings of empathy are subject to context^4^, the recipient’s identity^5^, and how culpable the victim is perceived to be^6^. For example, individuals feel less empathy for others receiving painful injections when they’re told it is due to their own life choices versus an illness outside their control^6^. Other factors, like personal experience also may come into play^7^. For example, victims of sexual assault are more empathetic to others who have endured a similar experience^8^. This effect is explained by the theory of “altruism born of suffering”^9^, which posits that individuals are more willing to help others in situations they have experienced themselves. By experiencing an event, an individual is better able to place themselves in the same shoes as another and imagine the other person’s emotions^9^.

While many studies investigated the effect of previous experience (e.g. rape^8^, social rejection^10^, loss of a pet^11^), relatively little work has been done on more generalized maltreatment, like early life adversity (ELA). ELA refers to extreme and/or chronic stress in childhood^12^, and is experienced in varying degrees by individuals. Some individuals report virtually no maltreatment, while others report having experienced multiple forms of abuse and neglect^13^. Often neglect and abuse are concomitantly experienced, as trying to separate the two is akin to “trying to pull apart a grey sweater in search of separate black threads and white threads”^13^.

Such experiences during childhood have profound effects on the brain and periphery, which become sculpted to expect a stressful and dangerous environment in adulthood, resulting in chronically “on guard” systems^14^, from the hypothalamic–pituitary–adrenal (HPA) axis^15,16^ to the immune system^17^. This increased sensitivity to negative environmental stimuli orients the individual towards perceiving threat and increases the risk for chronic stress^18–20^. Furthermore, ELA has profound effects on socio-emotional development (Aber and Cicchetti^21^) which is thought to arise due to the affected development of central neural circuits^18–20^. Adults who experienced ELA are more sensitive to negative stimuli in their environments^22,23^ have less connectivity between the amygdala and prefrontal cortex^24–26^, and this decreased connectivity has been reported to mediate the link between childhood maltreatment and depression and anxiety symptoms^26,27^. Furthermore, experiences of childhood maltreatment have been related to impaired cognitive empathy and lower empathic concern^28,29^. Additional work has found links between childhood maltreatment and adult empathy levels, yet these works were within the context of investigating borderline personality disorder^30^ and depression^31^.

While the profound psychophysiological effects of ELA cannot be discounted, the effects of ELA are not all negative, as it may lead to higher levels of resilience and compassion^32,33^. However, less is known about the connection between empathy and early maltreatment and neglect. Past research has mostly focused on child maltreatment and empathy in either childhood and adolescents^34,35^ or in clinical samples^30,31^, and not how empathy may be affected in a general adult sample. Given how past experiences can help increase empathy for others, perhaps experiences of ELA help individuals recognize the common humanity in negative or painful situations, and thus feel more empathy towards those experiencing them. Indeed, some recent work has linked ELA to elevated levels of empathy^36^, however with a few caveats. In said study, individuals who experienced at least one traumatic event in childhood had elevated levels of empathy compared to their no-trauma peers. However, due to the ubiquity of negative events in childhood, only 21% of their sample reported experiencing no traumatic events, and thus the “no trauma” group was the minority group, with most people falling in the “trauma” category. Classically, ELA is considered to be more chronic and intense to be considered the majority experience. The simultaneous use of different targeted questionnaires may better capture the spectrum of ELA that individuals report experiencing. Parental overprotection before the age of sixteen has also been related to increased levels of personal distress, however, researchers did not investigate whether maternal or paternal overprotection was driving this effect^37^.

In the following work, we wanted to probe how increasing levels of childhood maltreatment and neglect are related to empathy. Specifically, we sought to measure ELA experiences, and assess how they relate to various forms of cognitive and affective empathy. To investigate the dose-dependent association between ELA and empathy, we assessed ELA with the Childhood Trauma Questionnaire (CTQ), and the Parental Bonding Instrument (PBI) for mother and father in an online study. Empathy was assessed using the Interpersonal Reactivity Index (IRI) as well as willingness to donate a certain percentage of study compensation to a charity (donation rate) as an additional exploratory measure of prosocial behavior. Building on the work of Greenberg et al.^36^, we hypothesized a positive correlation between ELA and empathy (i.e. higher scores on all CTQ subscales as well as lower care and higher overprotection on the PBI subscales being associated with higher IRI subscales and donation rates). Results from this study add to the sparse literature connecting ELA and empathy and elucidate the dose relationship between ELA and empathy, and hopefully spur more work to explore the link between past experiences and empathy.

## Results

Mean ELA and empathy in this sample are depicted in Table 1. Furthermore, a group comparison for high vs. low ELA based on maternal care cut-off, as well as all data and analysis scripts used here can be found in the supplemental material (Supplemental Table 1) and at https://osf.io/d39pt/.

**Table 1:** Descriptive data for early life adversity and empathy.

	Total sample
	(*N* = 228)
CTQ	
Emotional abuse	12.29 (± 6.21)
Physical abuse	7.78 (± 4.52)
Sexual abuse	7.85 (± 5.78)
Emotional neglect	12.58 (± 5.94)
Physical neglect	8.74 (± 4.09)
PBI	
Mother (N = 191)	
Care	24.50 (± 9.42)
Overprotection	13.38 (± 9.52)
Father (N = 180)	
Care	21.34 (± 9.39)
Overprotection	11.12 (± 8.81)
IRI	
Empathic concern	3.82 (± 0.66)
Fantasy scale	3.60 (± 0.87)
Personal distress	3.24 (± 0.88)
Perspective taking	3.67 (± 0.69)
Donation (N = 216)	2.71 (± 1.86)

Descriptive data are presented as mean (± standard deviation).

*CTQ* childhood trauma questionnaire, *PBI* parental bonding instrument, *IRI* interpersonal reactivity index.

**Figure 1:**
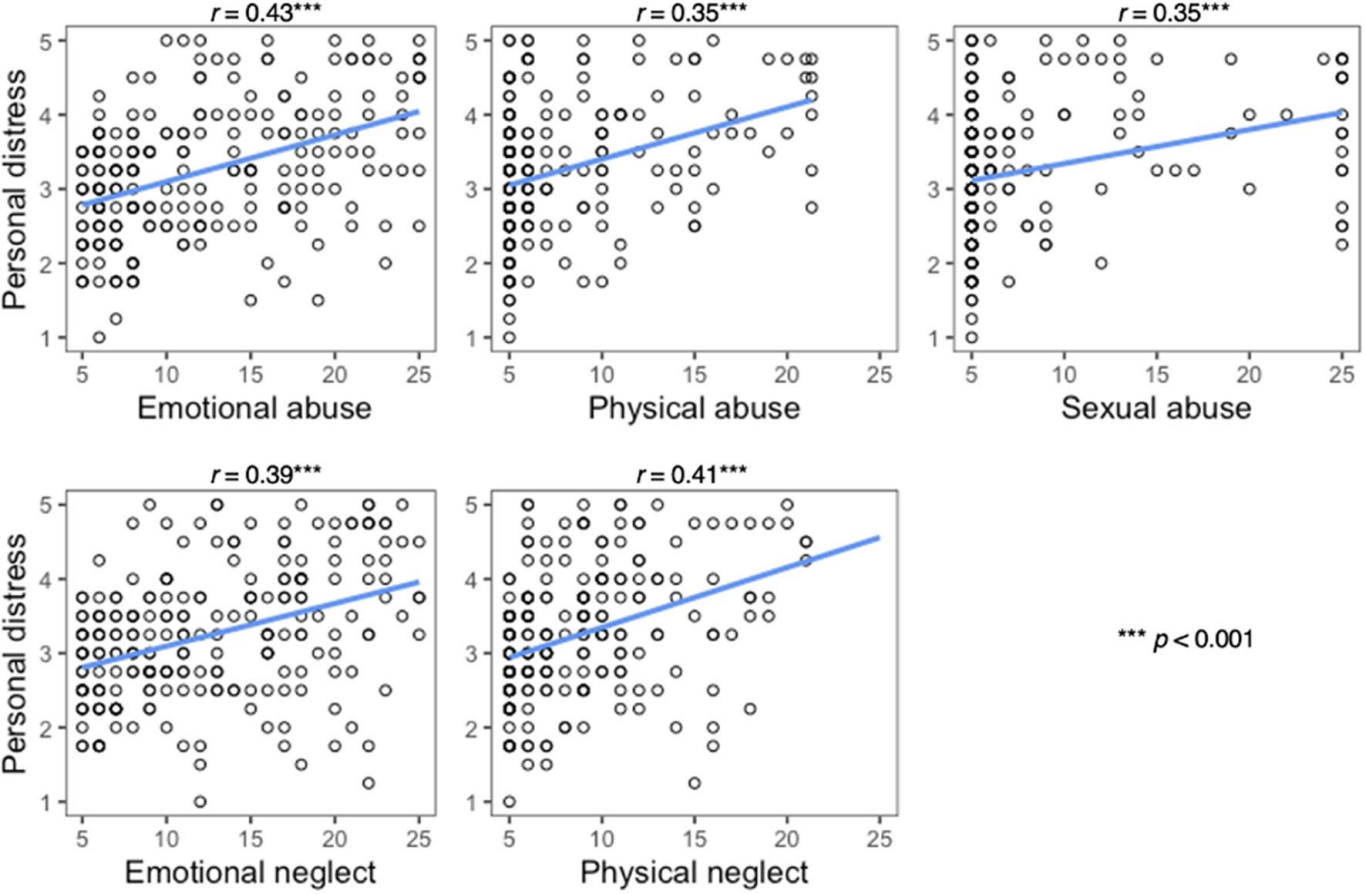
Association between CTQ (= Childhood Trauma Questionnaire) subscales and personal distress in response to others’ suffering.

**Figure 2:**
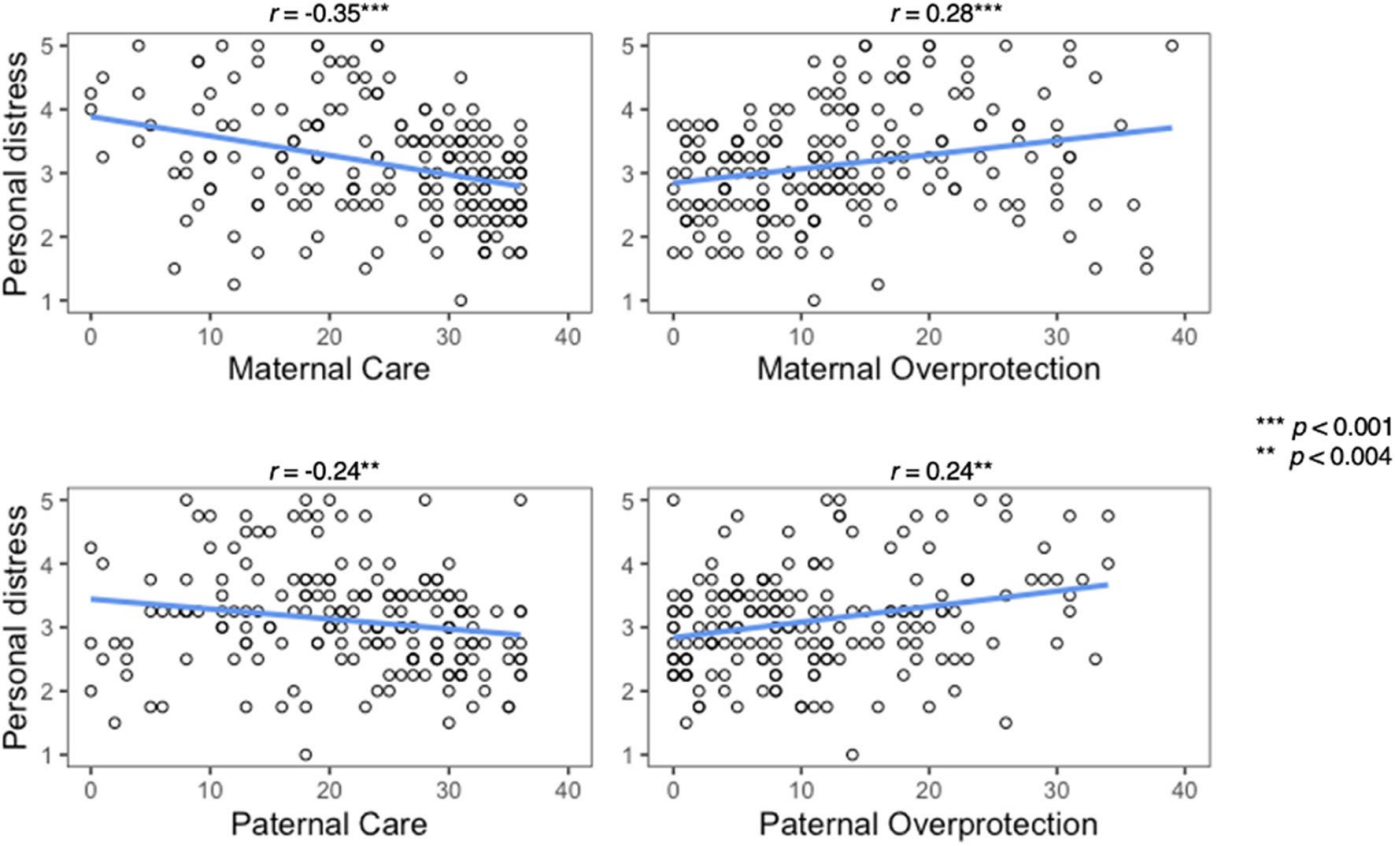
Association between PBI (= parental bonding instrument) subscales and personal distress in response to others’ suffering.

While we could not find any association between the donation rate and the IRI subscales (Donation ~ IRI: *r* =  − 0.06 to 0.10 for all, p > 0.05), the personal distress subscale of the IRI was correlated with all CTQ and PBI subscales (see Table 2, Supplemental Fig. 1). Participants who reported higher levels of abuse and neglect showed higher levels of personal distress in response to others’ suffering (see Fig. 1). Likewise lower parental care and higher parental overprotection were associated with higher personal distress (see Fig. 2).

**Table 2:** Spearman correlation coefficients.

	Empathy (IRI)				Donation
	Perspective taking	Empathetic concern	Personal distress	Fantasy scale	(n = 216)
CTQ					
Emotional abuse	− 0.06	− 0.01	0.43***	− 0.10	0.12
Physical abuse	− 0.07	− 0.03	0.35***	− 0.12	0.14*
Sexual abuse	0.02	0.10	0.35***	− 0.04	0.22**
Emotional neglect	< 0.01	− 0.02	0.39***	− 0.14*	0.14*
Physical neglect	− 0.03	0.02	0.41***	− 0.13	0.19*
PBI					
Maternal (*n* = 191)					(*n* = 183)
Care	0.02	0.01	− 0.35***	0.03	− 0.14
Overprotection	− 0.07	− 0.09	0.28***	− 0.07	0.06
Paternal (*n* = 180)					(*n* = 172)
Care	0.01	0.08	− 0.24**	0.14	− 0.08
Overprotection	− 0.04	− 0.09	0.24**	− 0.07	− 0.01

*CTQ* childhood trauma questionnaire, *PBI* parental bonding instrument, *IRI* interpersonal reactivity index.

Correlation CTQ ~ IRI based on *N* = 228, other correlations reduced according to reduced sample sizes due to missing values on the respective scale; significant correlations after Bonferroni correction are printed in bold.

***p < 0.001, **p < 0.004 (adjusted alpha level after Bonferroni correction for 14 variables), *p < 0.05.

Furthermore, participants who experience higher levels of sexual abuse tended to donate more at the end of the study. Beyond that, positive associations between donation rate and physical abuse, as well as emotional and physical neglect did not pass Bonferroni correction for multiple testing. Table 2 presents all results from Spearman correlation analyses. Adding age as a covariate did not change these effects. Additional analyses with sex and gender as covariates can be found in the supplemental material (Supplemental Tables 2, 3) and in our analysis script and output at https://osf.io/d39pt/ but will not be reported or discussed here due to severe group imbalance that limits interpretability.

Adding all five CTQ subscales to a stepwise regression to predict personal distress, we found only emotional abuse to explain additional variance of personal distress (F(1,226) = 55.29, p < 0.001, adjusted R^2^ = 0.19; see Table 3).

**Table 3:** Coefficients in the final model of forward stepwise regression to predict personal distress.

	Estimate	Std. error	t-value	p
(Intercept)	2.47	0.12	21.18	< 0.001
Emotional abuse	0.06	0.01	7.44	< 0.001

## Discussion

Looking at childhood maltreatment and empathy in this sample with a broad distribution of early life adversity, all indices of early life adversity were related to increased levels of personal distress in response to other’s suffering. Increased reported levels of emotional abuse, physical abuse, sexual abuse, emotional neglect, and physical neglect were all positively correlated with high levels of personal distress, as measured with the IRI. Of these, emotional abuse was most explanatory for differences in personal distress. Perhaps this may be explained by the relationship between emotional abuse and emotional dysregulation; Ref.^38^ found emotional abuse to be the strongest predictor of emotional dysregulation (over physical and sexual abuse) in an adult sample. Furthermore, higher reported maternal and paternal overprotection, and lower maternal and paternal care, were also related to higher levels of personal distress. In a recent study, parental overprotection was related to increased levels of personal distress, which is in line with the current findings^39^. Parental care and overprotection affect emotion regulation abilities; individuals with poor perceived parental care and high parental overprotection tend to have trouble with impulse control and acceptance of their emotions^40^. Such differences in regulation may affect one’s ability to regulate emotional responses to the distress of others, and thus, lead to increases in personal distress. Overall, these results support a linear positive relationship between negative childhood experiences and feelings of personal distress in adulthood. Of note, the findings suggest that high levels of trauma are not necessarily driving the effect; perceptions of parental care were also related to personal distress, which highlights how personal distress may increase with incremental decreases in perceived parental care. While participants with higher levels of ELA generally donated more of their study compensation to a charity, only higher levels of sexual abuse were significantly related to larger donations after correction for multiple statistical tests.

Other facets of empathy measured by the IRI (empathic concern, perspective taking, and fantasy) were not found to be related to any other ELA measure, which suggests ELA only affects levels of personal distress. To disentangle this finding, we must look at empathy by its main components, which are affective and cognitive empathy^41^. Personal distress and empathic concern are both separate types of affective empathy, with the former being a self-oriented response and the latter being a more other-oriented response to feelings elicited by the target^42^. Perspective taking and fantasy are both types of cognitive empathy, which involve adopting the point of view of someone else, whether in real life or in fantasy (e.g. movies, books^43^. Thus, ELA was not related to levels of cognitive empathy, but only self-oriented affective empathy. In other words, childhood maltreatment affects how adults respond to empathy-inducing situations, in that it induces more negative feelings in those who experienced more ELA compared to those who did not. Similar findings have been observed in individuals with posttraumatic stress disorder, which suggests that experiencing traumatic events, generally, may increase the likelihood of feeling more personal distress on behalf of others^44^.

While personal distress is a subtype of empathy, it is not related to higher levels of helping behavior^45^. Since personal distress refers to the negative feelings one experiences when seeing another in distress, it is more likely to motivate withdrawal from the situation and interfere with the ability to provide help^46^. For example, if seeing a friend in pain, it is important to be able to manage and downregulate one’s own negative feelings in order to properly attend to and help one’s friend. Indeed, experiments have shown that while personal distress leads to faster responses to an emergency when alone with the victim, it is also related to a large decrease in action preparation when bystanders are present^47^. In the current study, higher levels of personal distress did not relate to giving a higher donation.

Despite the positive connotations of empathy, higher levels of personal distress do not necessarily translate to higher effective empathic behaviors. As discussed above, personal distress is not related to more helping behavior and, in fact, can hinder it. Increased levels of negative emotions can lead to egotistical motivations to withdraw instead of engage in prosocial behavior^46^. In therapeutic situations, therapists with more self-rated personal distress rated themselves as providing decreased quality in patient care^48^. Similarly, in physicians, personal distress appears to be negatively related to patient care^49^. Furthermore, levels of personal distress are related to depressive symptoms and alexithymia^50^. Due to the self-oriented negative emotions related to personal distress, it is thought that individuals who experience more personal distress generally experience more negative emotions, have less self-regulative control, and worse coping skills^51^.

Some limitations must be discussed when considering these results. Firstly, the following results are correlational and not causal. While it seems most logical to infer higher ELA leads to personal distress and not vice versa, due to ELA happening in childhood, we must consider the option that higher levels of personal distress may affect one’s retrospective judgments of childhood. In other words, perhaps individuals who experience more personal distress in response to empathy-inducing situations are more likely to have a negative affective veil over their childhood memories. Furthermore, questionnaire measures of ELA are imperfect assays of negative childhood experiences, as they are subject to present affective influences, and not objective assessments of actual experiences^13^. Likewise, the exploratory variable “donation” that we added as a behavioral measure is not validated and should be interpreted with some caution. In addition, most of the subscales that we used to operationalize ELA show medium to high inter-correlations^52^ which might lead to multicollinearity, especially limiting the interpretation of the stepwise regression. Further, we did not exclude participants based on any psychopathologies they may have had (as we wanted a representative sample of adults with experiences of ELA), and cannot discount the potential effect of psychopathology on these results. Finally, our sample was mostly female and well-educated, and it is unclear if these results are generalizable beyond this group.

Continued work is needed to gain a more comprehensive understanding of the link that exists between ELA and empathy. Future research can delve into the effects of ELA on psychophysiological responses to observing others in distress. This type of study would allow for more real-time objective measures of emotional responses to a target. Furthermore, more ecologically valid prosocial behaviors can be used^53^ to better assess how ELA affects the propensity to help others. Alternative assessments for ELA can also be used, such as objective reports from childhood (e.g. child protective services reports, arrest records of parents) and interviews. Work should be attentive to sex and gender differences and allow for more balanced distributions of participants to investigate differential effects of sex and gender on experiences of ELA.

Experiences of ELA imprint on the neuraxis and affect how individuals perceive and interact with their environments well into adulthood. The study presented here suggests that ELA may also be related to empathy, specifically the experience of personal distress. Thus, negative childhood memories are associated with having more feelings of personal distress when seeing others in distress. While this may not be related to explicit positive outcomes, like increased prosocial behavior, and in fact may be related to increased depressive symptoms, it nonetheless reflects how negative experiences may be related to how individuals react and feel when seeing others in distress. Our human experience involves a constant barrage of emotional triggers, and while it is important to self-regulate one’s emotions to not become overwhelmed, it is also important to retain the ability to feel, and feel the pain of others.

## Methods

### Participants

Participants were recruited online, through online trauma forums and via a study participant database provided by the University of Konstanz (SONA). Data collection took place from July to August 2021. Participants were required to be at least 18 years old, have sufficient German language skills, sign the informed consent, and agree to open access publication of de-identified data. Out of *N* = 280 data entries, 52 participants had to be excluded due to incomplete study participation or exclusion criteria fulfillment, resulting in a final sample of *N* = 228 (83% female, mean age = 30.51 ± 9.88 years, range 18–60). The sample characteristics are listed in Table 4. A-priori power analysis for correlation models using G*Power (Faul et al.^54^) with a medium effect size of 0.3, an alpha of 0.05, and a power of 0.95 revealed a minimum sample size of *N* = 111. Data collection was thus terminated after four weeks with a more than sufficient response rate.

**Table 4:** Sample characteristics.

	Female (*n* = 189)	Male (*n* = 39)	Total (*N* = 228)
Gender^a^			
Mean ± SD	4.46 ± 1.42	− 4.10 ± 2.33	2.99 ± 3.61
Range	− 5 to 5	− 5 to 5	− 5 to 5
Age			
Mean ± SD	30.61 ± 9.82	30.05 ± 10.28	30.51 ± 9.88
Range	18–60	19–55	18–60
Highest education level			
Lower than university-qualification	14%	15%	14%
Abitur (university-entrance level in Germany)	27%	46%	30%
Vocational training	17%	8%	15%
University degree (Bachelor, Master, PhD)	42%	31%	41%
In a relationship	59%	59%	59%

Sample characteristics are presented separated by self-reported sex assigned at birth with four response options (female, male, divers, no answer).

^a^Gender identification was assessed on a scale from − 5 = very masculine to + 5 = very feminine.

### Procedure

For data collection, we created an online survey using the online questionnaire platform Qualtrics (https://www.qualtrics.com). The survey took between 15 and 20 min to complete, for which participants could receive €5 Amazon vouchers as compensation. After participants provided information on demographic data, they completed self-report questionnaires on childhood adversity, parenting style, and empathy. In order to complement the self-reported empathy, an additional behavioral measurement was implemented, in which participants were given the opportunity to donate a part of their compensation to a German charity (Deutsche Welthungerhilfe e.V.). To ensure completion of all questionnaires, participants received an automated notification from Qualtrics reminding (but not forcing) participants to answer any remaining blank questions. The study protocol was approved by the Ethics Committee of the University of Konstanz and followed the guidelines outlined in the Declaration of Helsinki.

### Questionnaires

#### Early life adversity

To assess childhood adversity, the German version of the Childhood Trauma Questionnaire (CTQ, Bernstein et al.^55^) was applied. The CTQ consists of 28 items, with each item being rated on a 5-point Likert scale. It compromises five subscales based on item sum scores that operationalize different facets of abuse and neglect (emotional abuse, physical abuse, sexual abuse, emotional neglect, and physical neglect). Higher scores on the Likert-scale indicate higher levels of childhood adversity. The CTQ has shown very good reliability and validity ratings as well as a high agreement with the assessments of psychotherapists (Häuser et al.^56^). In the supplements (https://osf.io/d39pt/), internal consistency (Cronbach’s alpha) for the CTQ and all other questionnaires used in this study are depicted for this sample and from the literature (see Supplemental Table 4).

In addition to the CTQ, we used the Parental Bonding Instrument (PBI, ^57^) to retrospectively assess self-reported attachment aspects of perceived parenting behavior of mothers and fathers respectively. 25 items per parent cover the two dimensions care and overprotection on a 5-point Likert-Scale. On the care dimension, higher sum scores indicate more loving and affectionate parenting, while on the overprotection dimension, higher sum scores indicate more controlling and constraining parenting. For both, the English original and the German translation of the PBI, good psychometric properties have been reported^58^. Despite medium to high correlations between the CTQ and the PBI, there are meaningful differences in the subscales, especially regarding parental overprotection^52,58^. Thus, we decided to use both questionnaires to meet the idea of a broad assessment of early life adversity^59^.

#### Empathy

Individual differences in empathy were measured using the German 16-item version of the Interpersonal Reactivity Index (IRI-SPF, ^60^). The IRI^61^ consists of the four subscales perspective taking, fantasy, empathic concern, and personal distress, all answered on a 5-item Likert-scale. The fantasy scale captures the tendency to put oneself in the emotional world of fictional characters in books or movies, while the perspective taking scale is intended to measure cognitive tendencies to adopt another’s psychological viewpoint. The empathic concern scale evaluates the concern about the welfare of others in distress, the personal distress scale, in contrast, is designed to evaluate self-focused feelings such as discomfort and anxiety when observing others suffering^60^, thus providing a measurement of an aversive emotional response. For each subscale, mean scores were computed, as instructed by the authors. A high score in one of the subscales reflects perception of higher empathic tendency in the respective subscale. The IRI exhibits good reliability and factorial validity^60^.

Complementing the self-report measure of empathy and prosocial behavior, we added an exploratory behavioral measure and provided participants with the opportunity to donate a portion of their study compensation to a charity that supports suffering people in Madagascar. For this purpose, the situation in Madagascar as well as the aims of the charity were described in detail. After reading the description, participants were asked how much, if any, of their compensation they were willing to donate. They could choose to donate between 0 and 100% (in increments of 25%) of their compensation, with responses being coded for the statistical analysis on a 5-point scale ranging from 1 (0% donated) to 5 (100% donated). After termination of data collection, we donated the resulting amount to the described charity.

### Statistical analysis

We imputed missing values with the item median if less than 20% were missing within one participant on one scale or coded them as “NA” otherwise. To correct for outliers, we winsorized values below or above 3 standard deviations from the mean.

We analyzed the data using R version 4.1.0^62^, RStudio version 1.1.463 ^62^ including the built-in package ‘stats’, and the packages ‘arsenal’^63^ for descriptive analyses, and ‘Hmisc’ for correlation analysis. Graphs were created using ‘ggplot2’^64^. The level of significance was set to an alpha level of 0.05, and Bonferroni correction for a total of 14 variables (5 CTQ subscales, 4 PBI subscales, 4 IRI subscales, and 1 donation variable) was applied adjusting alpha level to 0.004. An R Markdown file with the statistical analysis as well as the data can be found at https://osf.io/d39pt/.

To test associations between ELA and empathy, we computed a correlation matrix with CTQ, PBI, and IRI subscales and the donation variable using the function rcorr() from the Hmisc-package to calculate spearman correlation coefficients to account for non-normal distributions of ELA variables. Additionally, based on the correlational results, we computed a forward stepwise regression using the built-in function step() adding all CTQ subscales (higher correlation coefficients than PBI subscales) to predict the IRI subscale “personal distress” (only IRI subscale that correlated with ELA).

## Supplementary Information

Supplementary Figure 1.

Supplementary Table 1.

Supplementary Table 2.

Supplementary Table 3.

Supplementary Table 4.

## Data Availability

The dataset generated and analyzed in the course of these studies, and the scripts of the statistical analysis are available online at https://osf.io/d39pt/ (Open Science Framework project 10.17605/OSF.IO/D39PT). We confirm that we report all data exclusions and all experimental manipulations.
