# Supplementary figures and images for: Increased empathic distress in adults is associated with higher levels of childhood maltreatment
Source: Sci Rep. 2023 Mar 11;13:4087. doi: 10.1038/s41598-023-30891-7 (PMC10008534; doi:10.1038/s41598-023-30891-7)

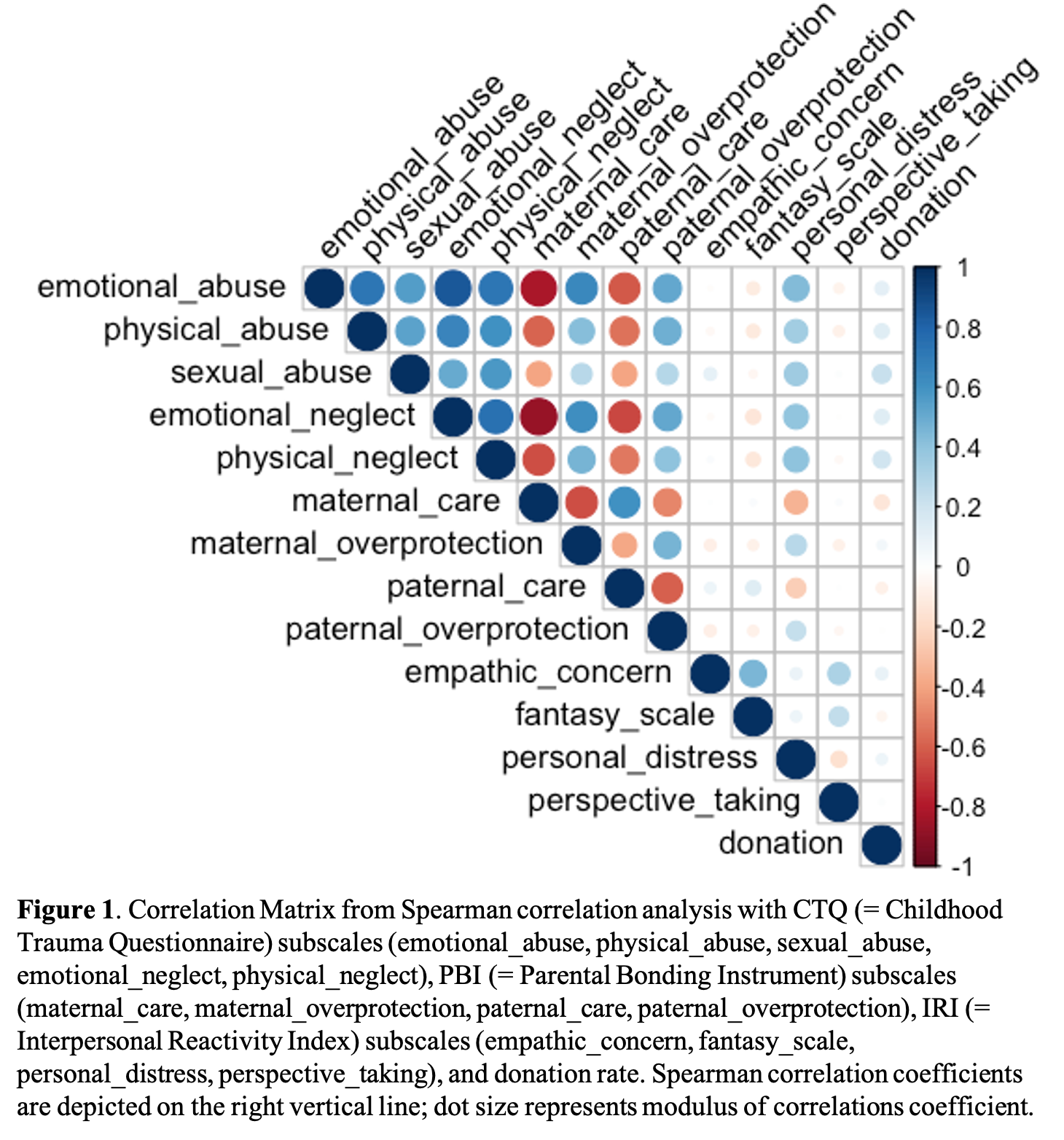

Supplement: Supplementary file 1 — Supplementary Figure 1. [file 41598_2023_30891_MOESM1_ESM.png]
