# Supplementary material for: Increased empathic distress in adults is associated with higher levels of childhood maltreatment
Source: Sci Rep. 2023 Mar 11;13:4087. doi: 10.1038/s41598-023-30891-7 (PMC10008534; doi:10.1038/s41598-023-30891-7)
Supplement: Supplementary file 2 — Supplementary Table 1. [file 41598_2023_30891_MOESM2_ESM.docx]

*Supplemental Table 1*. Sample characteristics and descriptive data for early life adversity and empathy

|  | **Total Sample** |  | **Maternal Care** | |  | **Group comparison** |
| --- | --- | --- | --- | --- | --- | --- |
|  | *(N = 228)* |  | High *(n = 99)* | Low *(n = 92)* |  | p-value |
| Sex ^a^ |  |  |  |  |  | .423 |
| Female | 189 (83%) |  | 83 (84%) | 156 (82%) |  |  |
| Male | 39 (17%) |  | 16 (16%) | 35 (18%) |  |  |
| Gender ^b^ | 2.99 (±3.61) |  | 3.22 (±3.5) | 2.73 (±3.80) |  | .350 |
| Age | 30.51 (±9.88) |  | 26.08 (±6.67) | 31.82 (±10.09) |  | < .001 |
| Education |  |  |  |  |  | .006 |
| High school graduation | 33 (15%) |  | 9 (9%) | 13 (14%) |  |  |
| University entrance level | 68 (30%) |  | 42 (42%) | 22 (24%) |  |  |
| Vocational training | 35 (15%) |  | 6 (6%) | 18 (20%) |  |  |
| Bachelor’s degree | 50 (22%) |  | 25 (25%) | 21 (23%) |  |  |
| Master’s degree or higher | 42 19%) |  | 17 (17%) | 18 (20%) |  |  |
| Relationship status (single, *N = 222*) | 87 (38%) |  | 42 (42%) | 33 (36%) |  | .215 |
|  |  |  |  |  |  |  |
| CTQ |  |  |  |  |  |  |
| Emotional abuse | 12.29 (±6.21) |  | 7.17 (±2.12) | 15.35 (±5.29) |  | < .001 |
| Physical abuse | 7.78 (±4.52) |  | 5.31 (±0.87) | 8.65 (±4.76) |  | < .001 |
| Sexual abuse | 7.85 (±5.78) |  | 5.37 (±1.36) | 8.62 (±6.38) |  | < .001 |
| Emotional neglect | 12.58 (±5.94) |  | 7.38 (±2.31) | 11.34 (±5.47) |  | < .001 |
| Physical neglect | 8.74 (±4.09) |  | 6.32 (±1.94) | 7.83 (±3.30) |  | < .001 |
|  |  |  |  |  |  |  |
| PBI |  |  |  |  |  |  |
| Mother | *N = 191* |  |  |  |  |  |
| Care | 24.50 (±9.42) |  | 32.08 (±2.71) | 16.34 (±6.90) |  | < .001 |
| Overprotection | 13.38 (±9.52) |  | 7.74 (±5.68) | 19.46 (±9.08) |  | < .001 |
| Father | *N = 180* |  | *n = 96* | *n = 67* |  |  |
| Care | 21.34 (±9.39) |  | 26.66 (±6.31) | 14.94 (±8.99) |  | < .001 |
| Overprotection | 11.12 (±8.81) |  | 6.65 (±5.72) | 15.51 (±8.86) |  | < .001 |
|  |  |  |  |  |  |  |
| IRI |  |  |  |  |  |  |
| Empathic concern | 3.82 (±0.66) |  | 3.85 (±0.60) | 3.85 (±0.74) |  | .953 |
| Fantasy scale | 3.60 (±0.87) |  | 3.68 (±0.82) | 3.63 (±0.88) |  | .690 |
| Personal distress | 3.24 (±0.88) |  | 2.87 (±0.65) | 3.44 (±0.96) |  | < .001 |
| Perspective Taking | 3.67 (±0.69) |  | 3.71 (±0.72) | 3.69 (±0.66) |  | .865 |
| Donation (N = 216) | 2.71 (±1.86) |  | 2.52 (±1.77) | 2.74 (±1.87) |  | .409 |

*Note*

Descriptive data are presented as n (%) for categorical and m (±sd) for metric variables;

p-values are reported for group comparisons based on Chi^2^-Tests or ANOVAs depending on data properties; we divided the two groups using the cut-off value <27 for maternal care proposed by Parker et al. (1979). CTQ = Childhood Trauma Questionnaire, PBI = Parental Bonding Instrument, IRI = Interpersonal Reactivity Index.

^a^ self-reported sex as assigned at birth, with the response options “female”, “male”, “diverse”, *n*=0 answered “diverse”, thus only two categories (female/male) are reported.

^b^ self-reported gender assessed on a scale from -5 = very male to +5 = very female.
