# Supplementary material for: Increased empathic distress in adults is associated with higher levels of childhood maltreatment
Source: Sci Rep. 2023 Mar 11;13:4087. doi: 10.1038/s41598-023-30891-7 (PMC10008534; doi:10.1038/s41598-023-30891-7)
Supplement: Supplementary file 3 — Supplementary Table 2. [file 41598_2023_30891_MOESM3_ESM.docx]

**Supplemental Table 2.** Regression coefficients for the association between the IRI fantasy scale and all CTQ and PBI subscales including sex as a covariate.

|  |  | **Estimate** | **Std. Error** | **t** | **p** |
| --- | --- | --- | --- | --- | --- |
| *CTQ* |  |  |  |  |  |
| Emotional abuse | (Intercept) | 3.89 | 0.13 | 29.99 | < .001 |
|  | Emotional Abuse | -0.02 | 0.01 | -2.13 | .034 |
|  | Sex (male) | -0.28 | 0.15 | -1.84 | .067 |
| Physical Abuse | (Intercept) | 3.85 | 0.12 | 32.25 | < .001 |
|  | Physical Abuse | -0.03 | 0.01 | -1.98 | .049 |
|  | Sex (male) | -0.27 | 0.15 | -1.82 | .070 |
| Sexual Abuse | (Intercept) | 3.81 | 0.10 | 37.46 | < .001 |
|  | Sexual Abuse | -0.02 | 0.01 | -204 | .043 |
|  | Sex (male) | -0.29 | 0.15 | -1.95 | .053 |
| Emotional Neglect | (Intercept) | 3.90 | 0.13 | 29.03 | < .001 |
|  | Emotional Neglect | -0.02 | 0.01 | -2.14 | .034 |
|  | Sex (male) | -0.25 | 0.15 | -1.65 | .100 |
| Physical Neglect | (Intercept) | 3.90 | 0.14 | 28.39 | < .001 |
|  | Physical Neglect | -0.03 | 0.01 | -2.05 | .041 |
|  | Sex (male) | -0.26 | 0.15 | -1.75 | .082 |
|  |  |  |  |  |  |
| *PBI* |  |  |  |  |  |
| Maternal Care | (Intercept) | 3.63 | 0.17 | 20.88 | < .001 |
|  | Maternal Care | <0.01 | 0.01 | 0.47 | .638 |
|  | Sex (male) | -0.27 | 0.16 | -1.73 | .086 |
| Maternal | (Intercept) | 3.79 | 0.11 | 35.21 | < .001 |
| Overprotection | Mat. Overprot. | -0.01 | 0.01 | -1.03 | .306 |
|  | Sex (male) | -0.26 | 0.16 | -1.66 | .100 |
| Paternal Care | (Intercept) | 3.42 | 0.16 | 21.81 | < .001 |
|  | Paternal Care | 0.02 | 0.01 | 2.34 | .021 |
|  | Sex (male) | -0.28 | 0.16 | -1.74 | .084 |
| Paternal | (Intercept) | 3.84 | 0.10 | 38.18 | < .001 |
| Overprotection | Pat. Overprot. | -0.01 | 0.01 | -1.17 | .244 |
|  | Sex (male) | -0.34 | 0.16 | -2.11 | .036 |

*Note*: Regression coefficients for lm(fantasy_scale ~ predictor + sex) are presented. Adding sex as a covariate to predict other IRI subscales did not change the outcome. Thus, those coefficients are only presented in the analysis output at https://osf.io/d39pt/. The question regarding biological sex was presented with three response options (female, male, divers), yet no one indicated “divers” as their sex, thus, this covariate compares female (n = 189) vs. male (n = 39) participants. IRI = Interpersonal Reactivity Index, CTQ = Childhood Trauma Questionnaire, PBI = Parental Bonding Instrument.
