# Supplementary material for: Increased empathic distress in adults is associated with higher levels of childhood maltreatment
Source: Sci Rep. 2023 Mar 11;13:4087. doi: 10.1038/s41598-023-30891-7 (PMC10008534; doi:10.1038/s41598-023-30891-7)
Supplement: Supplementary file 4 — Supplementary Table 3. [file 41598_2023_30891_MOESM4_ESM.docx]

**Supplemental Table 3.** Regression coefficients for the association between the IRI fantasy scale and all CTQ and PBI subscales including gender as a covariate.

|  |  | **Estimate** | **Std. Error** | **t** | **p** |
| --- | --- | --- | --- | --- | --- |
| *CTQ* |  |  |  |  |  |
| Emotional abuse | (Intercept) | 3.69 | 0.13 | 27.44 | < .001 |
|  | Emotional Abuse | -0.02 | 0.01 | -2.01 | .046 |
|  | Gender | 0.04 | 0.02 | 2.87 | .005 |
| Physical Abuse | (Intercept) | 3.67 | 0.12 | 29.35 | < .001 |
|  | Physical Abuse | -0.03 | 0.01 | -2.06 | .049 |
|  | Gender | 0.04 | 0.02 | 2.88 | .070 |
| Sexual Abuse | (Intercept) | 3.61 | 0.10 | 34.99 | < .001 |
|  | Sexual Abuse | -0.02 | 0.01 | -2.04 | .042 |
|  | Gender | 0.05 | 0.02 | 3.10 | .002 |
| Emotional Neglect | (Intercept) | 3.73 | 0.14 | 25.96 | < .001 |
|  | Emotional Neglect | -0.02 | 0.01 | -2.18 | .031 |
|  | Gender | 0.04 | 0.02 | 2.74 | .007 |
| Physical Neglect | (Intercept) | 3.71 | 0.14 | 25.79 | < .001 |
|  | Physical Neglect | -0.03 | 0.01 | -2.01 | .046 |
|  | Gender | 0.04 | 0.02 | 2.82 | .005 |
|  |  |  |  |  |  |
| *PBI* |  |  |  |  |  |
| Maternal Care | (Intercept) | 3.43 | 0.18 | 19.41 | < .001 |
|  | Maternal Care | < 0.01 | 0.01 | 0.63 | .531 |
|  | Gender | 0.04 | 0.02 | 2.30 | .022 |
| Maternal | (Intercept) | 3.63 | 0.12 | 30.20 | < .001 |
| Overprotection | Mat. Overprot. | -0.01 | 0.01 | -1.11 | .269 |
|  | Gender | 0.04 | 0.02 | 2.21 | .029 |
| Paternal Care | (Intercept) | 3.28 | 0.14 | 22.00 | < .001 |
|  | Paternal Care | 0.01 | 0.01 | 2.16 | .032 |
|  | Gender | 0.04 | 0.02 | 2.20 | .029 |
| Paternal | (Intercept) | 3.65 | 0.11 | 33.11 | < .001 |
| Overprotection | Pat. Overprot. | -0.01 | 0.01 | -1.12 | .265 |
|  | Gender | 0.04 | 0.02 | 2.64 | .009 |

*Note*: Regression coefficients for lm(fantasy_scale ~ predictor + sex) are presented. Adding gender as a covariate to predict other IRI subscales did not change the outcome. Thus, those coefficients are only presented in the analysis output at https://osf.io/d39pt/. Gender identification was assessed on a scale from -5 = very masculine to +5 = very feminine. IRI = Interpersonal Reactivity Index, CTQ = Childhood Trauma Questionnaire, PBI = Parental Bonding Instrument.
