# Supplementary material for: Increased empathic distress in adults is associated with higher levels of childhood maltreatment
Source: Sci Rep. 2023 Mar 11;13:4087. doi: 10.1038/s41598-023-30891-7 (PMC10008534; doi:10.1038/s41598-023-30891-7)
Supplement: Supplementary file 5 — Supplementary Table 4. [file 41598_2023_30891_MOESM5_ESM.docx]

**Supplemental Table 4.** Internal Consistency (Cronbach’s alpha) of all questionnaires

|  |  | Cronbach’s alpha | |
| --- | --- | --- | --- |
| Questionnaire | Subscale | Current sample *(N = 228)* | Original sample |
| CTQ | Emotional abuse | .93 | .89 |
|  | Physical abuse | .90 | .89 |
|  | Sexual abuse | .98 | .96 |
|  | Emotional neglect | .94 | .90 |
|  | Physical neglect | .73 | .62 |
| PBI | Maternal care | .95 | .95 |
|  | Maternal overprotection | .93 | .95 |
|  | Paternal care | .95 | .91 |
|  | Paternal overprotection | .92 | .92 |
| IRI | Empathic concern | .70 | .71 |
|  | Fantasy scale | .81 | .74 |
|  | Personal distress | .81 | .66 |
|  | Perspective taking | .76 | .71 |

*Note*: Cronbach’s alpha was calculated using the scoreItem()-function from the R package “psych” (Revelle, 2020); Cronbach’s alpha from the literature is reported for the respective German translation as cited in the original article. CTQ= Childhood Trauma Questionnaire, PBI = Parental Bonding Instrument, IRI = Interpersonal Reactivity Index.
